# Diagnostic and Prognostic Value of Long Noncoding RNAs as Potential Novel Biomarkers in Intrahepatic Cholestasis of Pregnancy

**DOI:** 10.1155/2021/8858326

**Published:** 2021-02-27

**Authors:** Shaohan Zou, Shaojie Zhao, Jing Wang, Ruirui Dong, Ping Zou, Fengbing Liang, Ting ting Zhu, Tao Zhou, Na Li, Yan Zhang, Tiejun Wang, Minjian Chen, Conghua Zhou, Ting Zhang, Liang Luo

**Affiliations:** ^1^Assisted Reproduction Unit, Department of Obstetrics and Gynecology, Sir Run Run Shaw Hospital, School of Medicine, Zhejiang University, Key Laboratory of Reproductive Dysfunction Management of Zhejiang Province, Hangzhou 310000, China; ^2^The Affiliated Wuxi Maternity and Child Health Care Hospital of Nanjing Medical University, Wuxi 214002, China; ^3^State Key Laboratory of Reproductive Medicine, Institute of Toxicology, School of Public Health, Nanjing Medical University, Nanjing 211166, China; ^4^Key Laboratory of Modern Toxicology of Ministry of Education, School of Public Health, Nanjing Medical University, Nanjing 211166, China; ^5^School of Computer Science and Telecommunication Engineering, Jiangsu University, Zhenjiang 212013, China; ^6^The Affiliated Wuxi No.2 People's Hospital of Nanjing Medical University, Wuxi 214002, China

## Abstract

Long noncoding RNAs (lncRNAs) are a class of important regulators participating in various pathological processes. Until now, the role of lncRNAs in the occurrence and development of intrahepatic cholestasis of pregnancy (ICP) has rarely been investigated. The data from microarray screening revealed 58 upregulated and 85 downregulated lncRNAs and 47 upregulated and 71 downregulated mRNAs in ICP patients compared to healthy controls. Bioinformatics analysis revealed biological processes focused on lipid metabolism, apoptosis, cell cycle, cell differentiation, and oxidative stress. Furthermore, the expressions of three lncRNAs (ENST00000505175.1, ASO3480, and ENST00000449605.1) chosen for verification were significantly decreased and showed the diagnostic and prognostic value for ICP based on ROC analysis. This is the first study to report the specific role of lncRNAs in ICP, which may be helpful for the diagnosis and prognosis of ICP clinically.

## 1. Introduction

Intrahepatic cholestasis of pregnancy (ICP) is distinguished by elevated liver enzymes and serum bile acids and pruritus [1, 2]. ICP patients are at a high risk of spontaneous fetal distress, sudden intrauterine death, and preterm labor [3]. Both genetic and environmental factors participate in the interpretation of the cause of ICP [4]. Several studies of ICP have revealed altered genes and proteins in many role places, including oxidative stress, cell growth, apoptosis, lipid metabolism, and immune responses [5, 6]. Currently, the identification of ICP is verified by symptoms and the serum level of total bile acids (TBA). However, the specificity is still limited. Therefore, the rapid diagnosis and treatment of ICP are important in obstetrics.

Long noncoding RNAs (lncRNAs) are transcripts with no protein-coding capability longer than 200 nucleotides [7]. During the last decade, they have been widely acknowledged as crucial regulators of various diseases, such as cancer, cardiovascular diseases, and rheumatoid diseases [8, 9]. They are involved in many pathophysiological and physiological processes, such as chromatin remodeling, cell cycle progression, posttranscriptional processing, and gene transcription [10, 11]. It has been reported that some lncRNAs are highly expressed in several types of cancers which makes them a promising tool for disease diagnosis. Some other lncRNAs are related to patient survival which makes them suitable for the prognosis of cancer [12]. Until now, the role and related mechanism of lncRNAs in ICP are still not fully elucidated. Hu et al. have found that linc02527 is upregulated in placenta and serum from ICP patients, and in vitro experiment using HTR8 cells reveals that linc02527 promotes autophagy and cell proliferation, which indicates that linc02527 may be a potential target for ICP treatment [6]. However, the relation of lncRNAs and the occurrence and development of ICP are still far from clear, clarifying that the assignment of lncRNAs in ICP may aid in understanding the pathogenesis of ICP and may supply new determinants for the diagnosis, treatment, and prognosis of ICP.

In our study, we investigated the expression profiles of lncRNAs using serum from ICP patients and explored the diagnostic and prognostic value of differently expressed lncRNAs in ICP.

## 2. Materials and Methods

### 2.1. Study Population

A case-control study was carried out to identify maternal serum lncRNAs for the diagnosis and prognosis of ICP. This study recruited 54 pregnant women with ICP and 54 healthy pregnant women from Wuxi Maternity and Child Health Care Hospital, Nanjing Medical University, between October 2016 and September 2017. No ursodeoxycholic acid was administered before blood sample collection. The criteria for diagnosing ICP and recruiting and excluding subjects were described previously [13]. ICP was diagnosed in women presenting with classical pruritus associated with liver dysfunction and raised serum total bile acids (TBA) (>10 *μ*mol/L as moderate ICP, >40 *μ*mol/L as severe ICP). All other causes of liver dysfunction, including preeclampsia, the HELLP (haemolysis, elevated liver enzymes, and low platelets) syndrome, acute fatty liver of pregnancy, primary biliary cirrhosis, viral hepatitis, and any ultrasound abnormality that may result in biliary obstruction were excluded. The characteristics of the patients and controls are in Table 1. All participants had signed written informed consent prior to recruitment, and the study protocol was approved by the Medical Ethics Committee of the Affiliated Wuxi Maternity and Child Health Care Hospital of Nanjing Medical University.

### 2.2. Sample Preparation

Peripheral blood (5 mL) was collected from each participant, and the serum was isolated within 4 h by centrifuge for 10 min at 4,000 rpm and then for 15 min at 12,000 rpm. The serum was removed carefully, divided into aliquots, and stored at −80°C. Total RNA was isolated by TRIzol (Invitrogen) and purified by a mirVana miRNA Isolation Kit (Ambion, Austin, TX, USA), in the light of the manufacturer's protocol. We determined the RNA concentration spectrophotometrically (NanoDrop ND-1000; Thermo Fisher Scientific, Waltham, MA, USA).

### 2.3. lncRNA Microarray

The global profiling of human lncRNAs and protein-coding transcripts was done with the CapitalBio Technology Human lncRNA Array v4. According to the manufacturer's standard protocols, the sample was prepared and microarray hybridization was done. We analyzed the acquired array images by Agilent Feature Extraction software (ver. 11.0.1.1). Summarization, standardization, and quality control of data were carried out by GeneSpring v13.0 (Agilent). Scatterplot filtering was used to identify those differentially expressed lncRNAs and mRNAs.

### 2.4. qPCR Validation

From 143 differentially expressed lncRNAs, we chose five respective lncRNAs with good consistency and small within-group and large between-group differences. Among these, RNA95791|RNS_873_113 and ENST00000503615.1 could not be detected in blood. Then, the expressions of another three lncRNAs were calculated by qRT-PCR (TaKaRa Bio, Inc., Tokyo, Japan) on an ABI 7500 (Applied Biosystems, Foster City, CA, USA). We normalized the expression level to miR-39 [14]. The primers used for qPCR of the lncRNAs are listed in Table 2. We used the 2−*ΔΔ*Ct method to calculate the relative expression of lncRNAs.

### 2.5. Bioinformatics Analysis

We got the mRNA and lncRNA microarray data from CapitalBio Corp. (Beijing, China). We selected the differentially expressed genes using thresholds of ≥2 and ≤-2 times variation. Further analyzation was done with hierarchical clustering with average linkage [15]. We use Gene Ontology (GO) analysis and pathway analysis to determine the potential function of maladjusted lncRNA and the role of differentially expressed mRNA.

### 2.6. Statistical Analysis

Statistical analyses were carried out by GraphPad 8.0 (GraphPad Software Inc., La Jolla, CA, USA) and SPSS 16.0 (SPSS Inc., Chicago, IL, USA). Data were performed as means ± SD for a minimum of three independent experiments in triplicate. Independent Student's *t*-test and one-way ANOVA were used to contrast different teams. We used ROC curve analysis to assess the ability of biomarkers to distinguish the disease group from the control group. A level of *P* < 0.05 was considered statistically significant.

## 3. Results

### 3.1. Screening and Identification of Differentially Expressed lncRNAs and mRNAs in ICP

**Figure 1:**
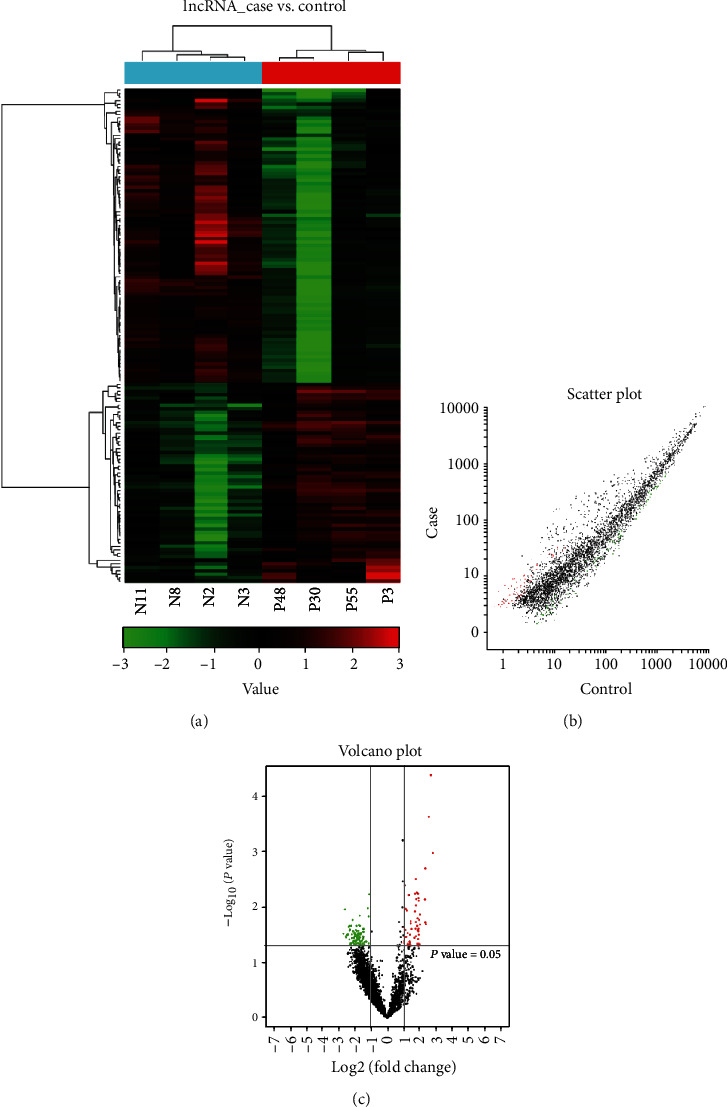
The lncRNA expression profile in ICP compared with controls. (a) Differently expressed lncRNAs by hierarchical clustering analysis. Red represents upregulated genes, green represents downregulated genes, and the difference is more than 2 times. (b) Different expression of lncRNAs by scatter plot. Red represents upregulated genes, green represents downregulated genes, and the difference is more than 2 times. (c) Different expression of lncRNAs by volcano plot. Red represents upregulated genes, green represents downregulated genes, and the difference is more than 2 times.

**Figure 2:**
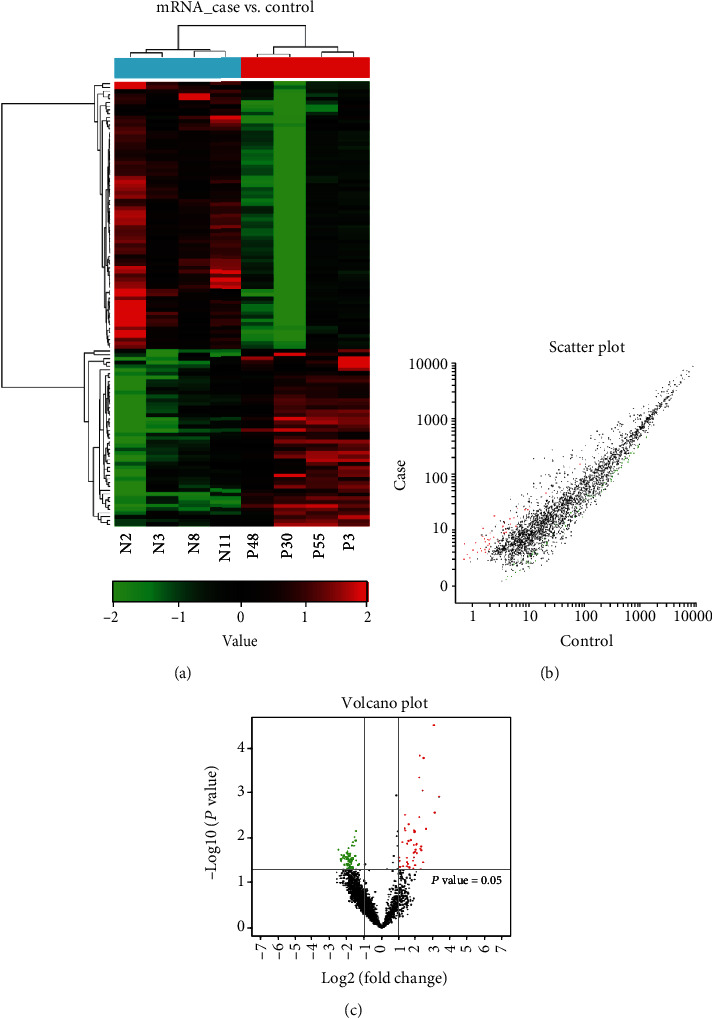
The mRNA expression profile in ICP compared with controls. (a) Differently expressed mRNAs by hierarchical clustering analysis. Red represents upregulated genes, green represents downregulated genes, and the difference is more than 2 times. (b) Different expression of mRNAs by scatter plot. Red represents upregulated genes, green represents downregulated genes, and the difference is more than 2 times. (c) Different expression of mRNAs by volcano plot. Red represents upregulated genes, green represents downregulated genes, and the difference is more than 2 times.

**Figure 3:**
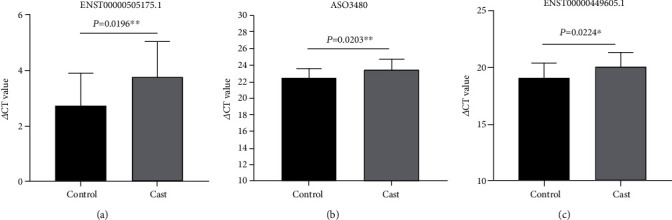
Real-time PCR confirmed three differentially expressed lncRNAs in 54 pairs of ICP patients and healthy controls. (a) ENST00000505175.1 was notably decreased in ICP compared with controls (^∗^*P* = 0.0196). (b) ASO3480 was markedly decreased in ICP compared with controls (^∗^*P* = 0.0203). (c) ENST00000449605.1 was obviously decreased in ICP compared with controls (^∗^*P* = 0.0224).

The expression profiles were compared from ICP patients and healthy controls using the CapitalBio Technology Human lncRNA Array v4. The hierarchical clustering analysis, scatterplot, and volcano plot from microarray screening showed that there were 58 upregulated and 85 downregulated lncRNAs, while 47 upregulated and 71 downregulated mRNAs in the ICP group compared to the HC group (Figures 1 and 2).  Table 3 shows the information of the aberrantly presented lncRNAs. From 143 differentially expressed lncRNAs, the 5 differentially expressed lncRNAs were chosen for verification by qRT-PCR in the serum, due to their expressions showing little internal difference within the groups but pronounced difference among the groups. Among these, RNA95791 and RNS_873_113 were not detected in the serum of the ICP group. Another three lncRNAs, ENST00000505175.1, ASO3480, and ENST00000449605, were significantly downregulated in the ICP group contrasted with the control (Figure 3).

### 3.2. Bioinformatics Analysis of Differentially Expressed lncRNAs and mRNAs in ICP

To find the possible enrichment of biofunctions of those differentially expressed lncRNAs and mRNAs, we did pathway analysis using Gene Ontology (GO) terms (Table 4). It showed that the significantly enriched terms include the cell cycle process, fatty acid metabolic process, apoptotic signaling pathway, and oxidative stress. Furthermore, the aberrantly expressed mRNAs in the ICP group are HAND1 (heart- and neural crest derivative-expressed protein 1), MRPS16, and AQP2 (Aquaporin 2). The results revealed the association of differentially expressed lncRNAs and mRNAs in ICP.

### 3.3. The Potential Diagnostic Value of Serum lncRNAs for ICP

**Figure 4:**
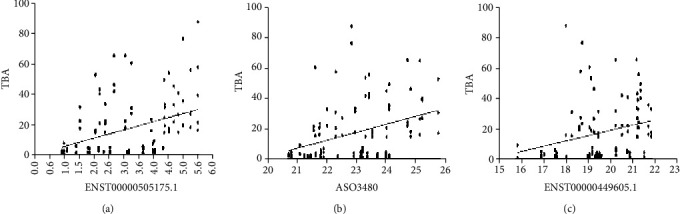
The correlation of serum lncRNA level and TBA in ICP patients. (a) ENST00000505175.1 (*R* = 0.401, *P* < 0.0001), (b) ASO3480 (*R* = 0.319, *P* = 0.0009), and (c) ENST00000449605.1 (*R* = 0.300, *P* = 0.0018).

**Figure 5:**
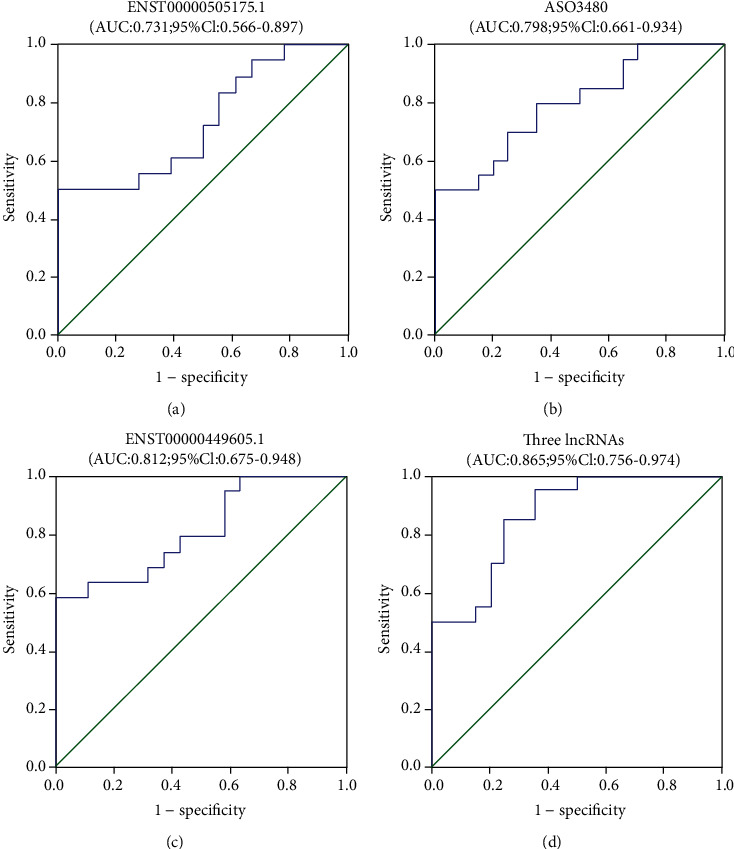
Diagnostic value of serum lncRNAs in ICP patients. (a) ENST00000505175.1, (b) ASO3480, (c) ENST00000449605.1, (d) ROC curve of the union of the 3 lncRNAs by multiple logistic regression analysis. The union of the 3 lncRNAs (ENST00000505175.1, ASO3480, and ENST00000449605.1) yielded the largest AUC.

To evaluate the diagnostic value of lncRNAs, the association between ENST00000505175.1, ASO3480, ENST00000449605.1, and TBA was analyzed using Pearson correlation analysis. It showed that the levels of ENST00000505175.1, ASO3480, and ENST00000449605.1 were positively associated with the TBA (*R* = 0.401, *P* < 0.0001; *R* = 0.319, *P* = 0.0009; and *R* = 0.300, *P* = 0.0018, respectively) (Figure 4). Furthermore, the sensitivity and specificity of lncRNAs for ICP diagnosis were evaluated. The ROC curves of ENST00000505175.1/ASO3480/ENST00000449605.1 show strong separation between ICP and non-ICP groups, with an area under the curve (AUC) of 0.731 (95% confidence interval 0.566–0.897) for ENST00000505175.1, 0.798 (95% confidence interval 0.661–0.934) for ASO3480, 0.812 (95% confidence interval 0.675–0.948) for ENST00000449605.1, and 0.865 (95% confidence interval, 0.756–0.974) for ENST00000505175.1/ASO3480/ENST00000449605.1, respectively (Figure 5). The results revealed such three lncRNAs have a diagnostic value for ICP, which can be a supplement for TBA diagnosis.

### 3.4. The Potential Prognostic Value of Serum lncRNAs for ICP

To evaluate the prognostic value of lncRNAs, the association between ENST00000505175.1, ASO3480, ENST00000449605.1, and perinatal outcomes of ICP was analyzed using the chi-squared test. The lncRNA expression was normalized by log2 transformation. We used the median serum lncRNA level as the critical value in the samples [16]. The group with low lncRNA expression had worse perinatal outcomes, such as meconium-stained amniotic fluid, fetal distress, and premature delivery (Table 5). The difference between the groups was significant. The results revealed that such three lncRNAs have a prognostic value for ICP, which can be a potential monitor for ICP treatment.

## 4. Discussion

Epidemiological studies indicate that the prevalence of ICP is increasing due to the change in eating habits and living environments. ICP can cause premature delivery, meconium-stained amniotic fluid, unexplained fetal death, and postpartum hemorrhage of pregnant women [17]. Therefore, an early, accurate diagnosis of ICP is essential. Currently, ICP is diagnosed using the serum TBA level; elevation of TBA is the most frequent laboratory abnormality associated with ICP and the most sensitive marker for ICP. However, the TBA level is normal in the ICP patients with low pruritus in some cases [18, 19]. Therefore, it is significant to find novel biomarkers as a supplement for TBA diagnosis.

Circulating lncRNAs caused increasing advertence as a novel diagnostic tool due to the relatively noninvasive nature of their gathering [20, 21]. Moreover, they are easy to amplify and not susceptible to denaturation or modification. For example, lncRNA HOTAIR in the serum/plasma of pancreatic cancer patients is a diagnostic and prognostic marker [22]. Zhang et al. have revealed that the lncRNA MALAT1 is a new biomarker for predicting *gestational diabetes* [23]. MALAT1 can also be used to predict metastasis and survival in patients with early-stage non-small-cell lung cancer [24]. A few of lncRNAs were verified in the human placenta, which is reported to regulate the response of the host to viral infection in human placental progenitor cells [25]. Yet, the expression and function of lncRNAs in the occurrence and development of ICP are still not clear. In this study, we found 143 differentially expressed lncRNAs using a CapitalBio Technology Human lncRNA Array v4 and identified three lncRNAs using qPCR. Bioinformatics technology revealed that target mRNA is mainly enriched in some processes such as cell cycle, fatty acid metabolism, apoptotic signaling, and oxidative stress [26–29]. According to the software prediction, the transcription factor of lncRNA ENST00000505175.1 is Oct-1 which participates in regulating a variety of physiological and pathological processes [30]. At present, studies have shown that Oct-1 is involved in the regulation of gastric cancer, colorectal cancer, ovarian cancer, and other cancers [31, 32]. In cancer, Oct-1 advanced the proliferation, migration, and invasion of cancer cells by regulating ERK and other signaling pathways [5, 33–35]. However, the detailed mechanism of such three lncRNAs would be further investigated.

Furthermore, the diagnostic and prognostic values of ENST00000505175.1, ASO3480, and ENST00000449605.1 in ICP were evaluated. We built ROC curves and computed the AUCs, which were 0.731, 0.798, and 0.812, respectively. Of note, the AUC (0.865) increased when we combined the three lncRNAs by multiple logistic regression analysis, indicating that the union of three lncRNAs is a more reliable ICP diagnostic marker. The prognostic significance of the serum lncRNAs in different risk subgroups based on meconium-stained amniotic fluid, fetal distress, and premature delivery was assessed, and such three lncRNAs appeared to be negative prognostic factors for ICP risk. The patients with higher serum concentrations of lncRNAs had worse pregnancy outcomes than those with lower serum concentrations especially when the three lncRNAs are used together, concluding that serum concentrations of the three lncRNAs could serve as a feasible prognostic biomarker of ICP.

However, our preliminary research only showed the differential expressions of these three lncRNAs in ICP patients and their potential significance. Whether such three lncRNAs can be finally applied to the clinic and a subgroup analysis between the patients with TBA levels ≥ 40 *μ*mol/L and TBA levels < 40 *μ*mol/L requires a large sample of data surveys and the related optimization designs. In addition, the functions of such three lncRNAs in ICP are still in progress, and further basic research is needed.

## 5. Conclusions

In summary, the serum level of the three lncRNAs may be used as the potential biomarkers of ICP. Combining the serum lncRNA levels with clinical markers commonly used in ICP, such as TBA, alanine aminotransferase, and glycocholic acid, may improve the diagnosis of ICP and merits further investigation.

## Figures and Tables

**Table 1:** The main clinical characteristics of the pregnant women with ICP and the healthy pregnant women.

Variable	Screening samples		*P* value	Validation samples		*P* value
	ICP (*n* = 4)	Control (*n* = 4)		ICP (*n* = 54)	Control (*n* = 54)	
Maternal age (y)	26.50 ± 3.87	27.2 ± 1.50	0.231	28.90 ± 6.32	26.1 ± 4.87	0.220
Gestational weeks	37.85 ± 0.77	38.72 ± 0.63	0.076	37.93 ± 0.93	38.92 ± 0.95	0.041
TBA (*μ*mol/L)	58.33 ± 12.4^∗^	3.51 ± 1.72	0.003	68.93 ± 50.35^∗^	5.34 ± 3.17	0.019
ALT (IU/L)	68.25 ± 24.76^∗^	17.01 ± 8.37	0.016	108.65 ± 101.44^∗^	16.1 ± 7.68	0.018
AST (IU/L)	82.71 ± 29.53^∗^	19.36 ± 5.42	0.028	106.78 ± 96.11^∗^	16.92 ± 8.34	0.018

TBA: total bile acid; ALT: alanine transaminase; AST: aspartate transaminase; statistical analysis was performed using *t*-tests; ^∗^*P* < 0.05 was considered significant.

**Table 2:** Primer sequences used in validation of lncRNAs.

lncRNA	Forward primer (5′-3′)	Reverse primer (5′-3′)
ENST00000505175.1	GGCCAGTGACCTTGACCTT	TTGCTGCCTCTTATGCTCAC
ASO3480	TTGATGGCTGGCAGTGCTC	CCATGTTGAGGCAGCACATC
ENST00000449605.1	CAGGCTGGGCAACATAGTGA	CCTGGGCTCAAACGATGCT
ST00000503615.1	GCCTGCGTGATTCTAGACTT	GACAGAGCGTCCACATTTTC
RNA95791|RNS_873_113	ATAAAGGGGATTCGGATGTC	TTTTTTAAACCCCTTAAGAACTAC

**Table 3:** Part of differentially expressed lncRNAs in the serum from pregnant women with ICP (P) compared to healthy pregnant women (C).

lncRNA ID	Fold change (P : C ratio)	*P* value	Regulation
*RNA95791|RNS_873_113*	6.96	1.06∗10^−3^	Up
ENST00000446102.1	6.40	4.29∗10^−5^	Up
ENST00000439804.1	4.08	2.06∗10^−2^	Up
ENST00000534653.1	5.87	2.40∗10^−4^	Up
ENST00000609910.1	5.15	2.04∗10^−2^	Up
ENST00000483023.1	4.96	7.27∗10^−3^	Up
*ENST00000503615.1*	2.70	1.78∗10^−2^	Up
ENST00000584829.1	0.153	3.02∗10^−2^	Down
ENST00000523759.1	0.164	1.11∗10^−2^	Down
TCONS_00011955	0.171	3.34∗10^−2^	Down
TCONS_00009146	0.240	3.56∗10^−2^	Down
*ENST00000449605.1*	0.242	4.57∗10^−2^	Down
HIT000248174	0.317	4.84∗10^−2^	Down
ENST00000604818.1	0.193	2.26∗10^−2^	Down
TCONS_00006708	0.338	4.93∗10^−2^	Down
ENST00000600160.1	0.266	2.17∗10^−2^	Down
*ASO3480*	0.267	4.58∗10^−2^	Down
ENST00000536898.1	0.198	3.84∗10^−2^	Down
HIT000430355	0.200	2.64∗10^−2^	Down
*ENST00000505175.1*	0.323	2.92∗10^−2^	Down

*P* is the probability of the difference of lncRNA expression between pregnant women with ICP (P) and healthy pregnant women (C). *P* < 0.05 was considered statistically significant. The chosen differentially expressed lncRNAs with verification by qRT-PCR were in italic.

**Table 4:** GO and pathway enriched analyses for differently expressed lncRNAs and mRNAs.

Category	Subcategory	*P* value	No. of genes	No. of target genes
Pathways	Primary bile acid biosynthesis	1.44∗10^−2^	36	6
	Oxidative stress	1.56∗10^−2^	69	15
	Fatty acid biosynthesis	2.06∗10^−2^	53	9
	Inflammation mediated by chemokine and cytokine signaling pathway	4.59∗10^−2^	77	15

GO: biological function	Response to fatty acid	5.81∗10^−3^	61	13

	Cell cycle	6.56∗10^−3^	61	15
	Neuron apoptotic process	8.41∗10^−3^	70	15
	Endothelial cell proliferation	1.22∗10^−2^	72	14
	Cell cycle process	1.46∗10^−2^	48	10
	Apoptotic DNA fragmentation	1.66∗10^−2^	63	14
	Mitotic cell cycle arrest	1.96∗10^−2^	63	12
	Adaptive immune response	2.04∗10^−2^	38	8
	Cell cycle checkpoint	2.19∗10^−2^	69	15
	Activation of cysteine-type endopeptidase activity involved in apoptotic process	2.71∗10^−2^	70	15
	Cell differentiation	2.86∗10^−2^	44	10
	Very long-chain fatty acid metabolic process	3.02∗10^−2^	29	6
	Innate immune response	3.22∗10^−2^	75	15
	Long-chain fatty acyl CoA biosynthetic process	3.25∗10^−2^	60	12
	Intrinsic apoptotic signaling pathway in response to endoplasmic reticulum stress	3.35∗10^−2^	57	13
	Extrinsic apoptotic signaling pathway	3.56∗10^−2^	54	9
	Oxidative stress	3.58∗10^−2^	67	14
	Cysteine-type endopeptidase activity involved in apoptotic process	3.78∗10^−2^	70	15
	Fatty acid metabolic process	3.86∗10^−2^	60	11
	Fatty acid biosynthetic process	4.12∗10^−2^	29	6
	Intrinsic apoptotic signaling pathway	4.72∗10^−2^	72	14

**Table 5:** The perinatal outcomes of human pregnant women.

Groups	*N*	Meconium-stained amniotic fluid (*N*)	*P* value	Fetal distress (*N*)	*P* value	Premature delivery (*N*)	*P* value
Control	54	8		6		3	
ENST00000505175.1 (<*M*)	20	3	0.984	2	0.891	3	0.186
ENST00000505175.1 (≥*M*)	34	9	0.177 0.328	10	0.03^∗^ 0.098	6	0.068 0.801
ASO3480 (<*M*)	27	7	0.23	6	0.185	3	0.368
ASO3480 (≥*M*)	27	12	0.004^∗∗^ 0.154	9	0.015^∗^ 0.362	9	0.001^∗∗^ 0.05
ENST00000449605.1 (<*M*)	27	7	0.225	3	1	4	0.162
ENST00000449605.1 (≥*M*)	27	12	0.004^∗∗^ 0.154	12	0.001^∗∗^ 0.001*^ΔΔ^*	8	0.003^∗∗^ 0.19
United lncRNAs (<*M*)	20	3	0.984	2	0.891	3	0.186
United lncRNAs (≥*M*)	34	16	0.001^∗∗^ 0.017*^Δ^*	13	0.003^∗∗^ 0.025*^Δ^*	9	0.005^∗∗^ 0.328

*M*: median value; *M* of ENST00000505175.1 = 4.356; *M* of ASO3480 = 4.517; *M* of ENST00000449605.1 = 4.348; *M* of united lncRNAs = 4.379; ^∗∗^*P* < 0.01 and ^∗^*P* < 0.05 vs. control groups; *^ΔΔ^P* < 0.01 and *^Δ^P* < 0.05 vs. <*M* groups.

## Data Availability

The datasets used and/or analyzed during the current study are available from the corresponding authors on reasonable request.
